# Dietary Methionine Intake and Risk of Incident Colorectal Cancer: A Meta-Analysis of 8 Prospective Studies Involving 431,029 Participants

**DOI:** 10.1371/journal.pone.0083588

**Published:** 2013-12-10

**Authors:** Zhong-Yin Zhou, Xin-Yue Wan, Ji-Wang Cao

**Affiliations:** Department of Gastroenterology, Renmin Hospital of Wuhan University, Wuhan, Hubei Province, China; Sookmyung Women's University, Korea, Republic Of

## Abstract

**Background:**

Methionine is one of the key components of one carbon metabolism. Experimental studies indicate that methionine may reduce inflammation-induced colon cancer. However, epidemiologic findings as to whether dietary methionine intake influences colorectal cancer incidence in humans are inconsistent.

**Objective:**

To investigate the relationship between dietary methionine intake and risk of colorectal cancer by performing a meta-analysis of prospective studies.

**Methods:**

Eligible studies were identified by searching PubMed and Embase and by reviewing the bibliographies of the retrieved publications. The summary risk estimates were computed using both a random- effects and a fixed-effects model.

**Results:**

Eight eligible prospective cohort studies involving 431,029 participants and 6,331 colorectal cancer cases were identified. According to the random-effects model, the summary relative risks (RRs) for the highest compared with the lowest intake of methionine were 0.89 (95% confidence interval [CI] = 0.77-1.03) for colorectal cancer, 0.77 (95% CI = 0.64 - 0.92) for colon cancer, and 0.88 (95% CI = 0.55-1.42) for rectal cancer. In the stratified analysis, a significant inverse association between dietary methionine intake and risk of colorectal cancer was observed in studies with longer follow-up time (RR=0.81, 95% CI= 0.70- 0.95), in Western studies (RR= 0.83, 95% CI = 0.73 - 0.95) and in men (RR = 0.75, 95% CI= 0.57-0.99). We found no indication of publication bias.

**Conclusion:**

This meta-analysis indicates that dietary methionine intake may be associated with decreased risk of colorectal cancer, especially colon cancer. More prospective studies with long follow-up time are needed to confirm these findings.

## Introduction

Colorectal cancer (CRC) is the third most common type of cancer worldwide, accounting for more than one million cases and 600 thousand deaths every year [1]. It is estimated that, in the United States alone, 26,470 deaths in males and 25, 220 deaths in females would occur in 2012 [2]. Screening and surveillance of adenomatous polyps, a precursor of colorectal cancer, is currently the cornerstone for primary prevention of colorectal cancer [3]. However, aside from the medical treatments for the risk factors of CRC, modifiable lifestyle factors are also widely considered to influence the development of this disease. 

One-carbon metabolism is characterized by integrated metabolic pathways centered on folate and methionine metabolism. The term one-carbon refers to one-carbon groups, such as methyl, methylene, methenyl and formyl [4,5]. One-carbon nutrients and their metabolites are involved in multiple biological processes, including synthesis of DNA, protein, amino acid and regulation of redox, etc. [4,5].

 Dietary folate intake has long been suggested to be associated with decreased risk of CRC, although the findings are not totally consistent [6]. Regarding the effect of dietary methionine intake on the development of CRC, current evidence is limited and inconclusive. It is widely accepted that cancer-associated inflammation promotes tumor growth and progression by means of inducing gene mutations, inhibiting apoptosis, and stimulating angiogenesis and cell proliferation [7,8], and methionine has been shown to be involved in the reduction of inflammation-induced colon cancer, and the inhibition of several pathways important in colon carcinogenesis [9-11]. Therefore, it is biologically plausible that dietary methionine intake might have a protective effect on CRC risk. 

To date, several epidemiologic studies examining the association between dietary methionine intake and risk of incident CRC have been carried out, but the findings are conflicting [12-18]. We conducted this meta-analysis of prospective cohort studies to quantitatively evaluate the relationship between dietary methionine intake and risk of incident CRC. 

## Materials and Methods

### Literature search

We performed a systematic search of PubMed (http://www.ncbi.nlm.nih.gov/pubmed) and Embase (http://www.embase.com) databases up to July 2013 by using the search terms *methionine* combined with *colorectal* or *colon* or *rectal*. No language restrictions were imposed. We also searched the reference lists of included publications to identify any further studies. Studies included in this meta-analysis had to meet the following criteria: the study design was prospective; the exposure of interest was dietary intake of methionine; the outcome of interest was incident CRC (or colon or rectal cancer); and the relative risks (RRs) or hazard ratios (HRs), with the corresponding 95 % confidence intervals (CIs) were reported. In case the same study was published in more than one article, we included the one containing the largest number of events. 

On the basis of these inclusion criteria, one study [19] was excluded because the exposure of interest in this study was combined intakes of folate, methionine and alcohol; nine papers [20-28] were excluded because they were duplicate publications of three other articles [12,16,17] with larger sample size. For one study that had two overlapping publications [16,25], the one with larger events were selected for use in the overall analysis, and the other one providing results for colon and rectal cancer was used in the subgroup analysis, as these data were not reported in the one with larger events.

### Data extraction

Two author (Z.-Y.Z. and X.-Y.W) independently collected the following characteristics for each study: the first author’s last name, publication year, study location, length of follow-up, number of CRC cases and participants, age, sex, the method used in methionine intake assessment, RR from the most fully adjusted model for the highest compared with the lowest methionine intake and the corresponding 95 % CI, and adjustment for potential confounder in a multivariate analysis. 

### Data synthesis and analysis

RRs or HRs were extracted from the selected publications, and their SEs were calculated from the respective CIs. The summary RRs with 95% CIs were calculated using both a fixed-effects model and a random-effects model [29]. Heterogeneity among studies was assessed with the Q and *I* ^2^ statistics [30]. For the Q statistic, we set the significance level at 0.10 instead of the more conventional level of 0.05, in order to avoid type II errors resulting from low power; for the *I* ^2^ statistic, a value of ＞50% was considered severe heterogeneity. 

Subgroup analyses according to years of follow-up, number of CRC cases, geographic location, sex, and subsites within colorectum were also performed to assess potential effect modification of these variables on outcomes. In addition, we also conducted a dose–response analysis by using the method proposed by Greenland and Longnecker [31] to compute study-specific slopes and 95% CIs from the natural logs of the RRs and CIs across categories of dietary methionine intake. For each study, the median or mean level of methionine intake for each category was assigned to each corresponding RR estimate. When the median or mean of per category was not provided in the article, we assigned to each class the dose corresponding to the midpoint of upper and lower boundaries. If the lower boundary of the lowest category or the upper boundary of the highest category was open, we considered them of the same amplitude as the closest category. Then, we obtained the summary RR estimates by pooling the study-specific slopes. 

Potential publication bias was evaluated with Begg’s funnel plots and Egger’s regression asymmetry test [32]. All analyses were performed using Stata version 12.0 (StataCorp, College Station, TX, USA). All statistical tests were two-sided.

## Results

**Figure 1:**
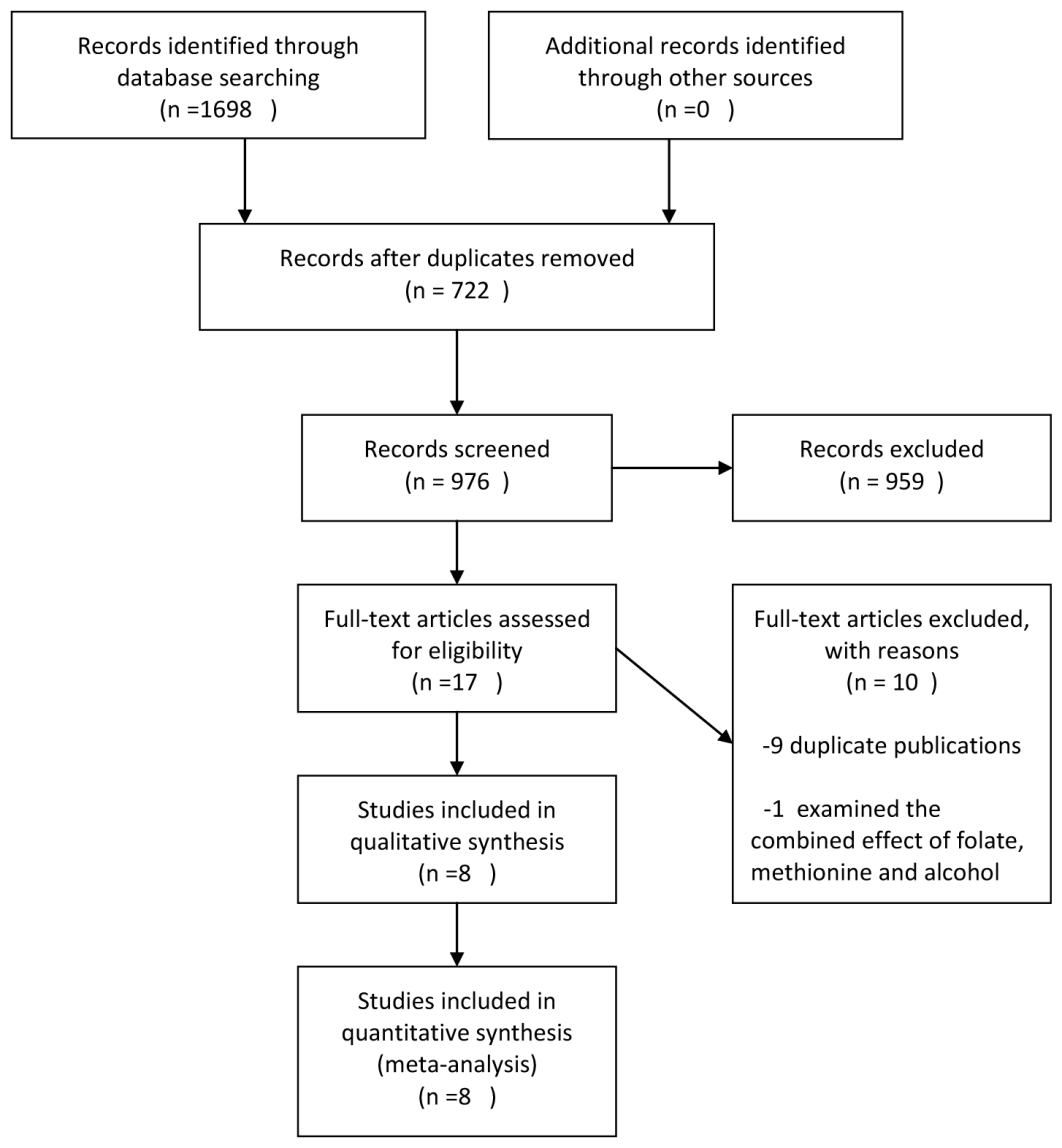
Flow chart of study selection.

Figure 1 presents the flow chart of literature search. Seven articles [12-18] containing eight prospective cohort studies (one article [17] had two independent cohorts) that investigated the relationship between dietary methionine intake and risk of CRC were included in the final analysis. The characteristics of these studies are shown in Table S1. The eight studies involved a total of 431,029 participants and 6,331 CRC cases, and were published between 2002 and 2012. These studies were carried out in the United States (*n* = 4), Canada (*n* = 1), Netherlands (*n* = 1), China (*n* = 1) and Japan (*n* = 1). The follow-up period ranged between 5.8 and 22 years. Diet including methionine intake was collected by either self-administered or interview-based food frequency questionnaire (FFQ). Most of included studies reported RRs (95% CIs) of CRC that were controlled for age (*n* = 7), body mass index (BMI) (*n* = 7), smoking (*n* = 7), physical activity (*n* = 5), family history of CRC (*n* = 4) and intakes of energy (*n* = 7), alcohol (*n* = 8), calcium (*n* = 7), meat (*n* = 6) and folate ( *n*= 4) (Table S1). 

**Figure 2:**
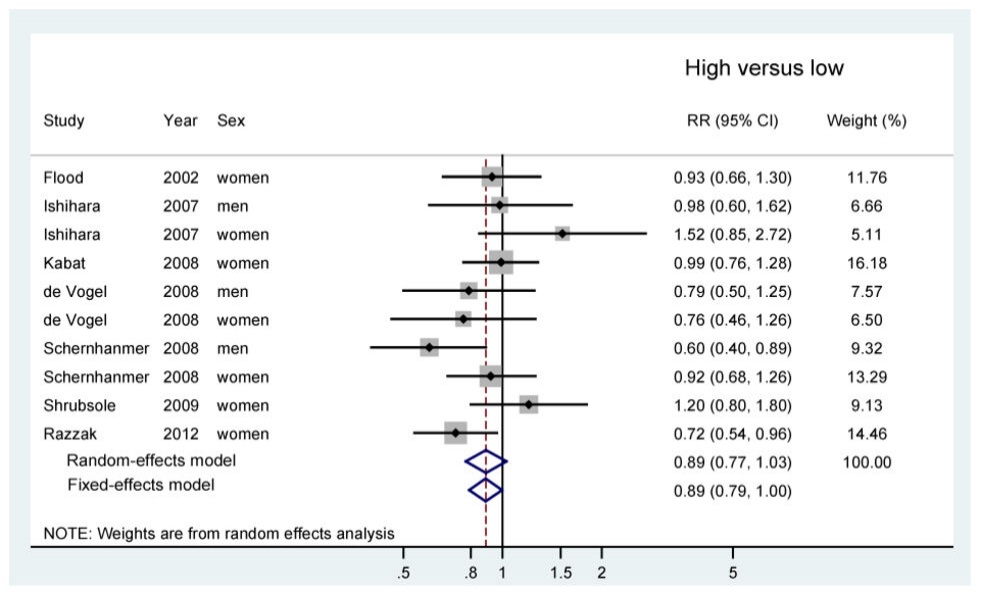
Forest plot showing relative risks of incident colorectal cancer for the highest compared with the lowest dietary methionine intake for individual studies and all studies combined. RR, relative risk; CI, confidence interval.

RR estimates of CRC for the highest compared with the lowest category of dietary methionine intake for individual studies and all studies combined are shown in Figure 2. The overall results suggested an inverse association of dietary methionine intake and risk of CRC, and the association was borderline significant according to the fixed-effects model (RR = 0.89, 95% CI = 0.79 - 1.00), but not random-effects model (RR = 0.89, 95% CI = 0.77 - 1.03). There was little evidence for heterogeneity (*P* for heterogeneity = 0.18, *I* ^2^ = 29.1%). 

Table 1 presents the results of subgroup analysis stratified by length of follow-up, number of CRC cases, geographic location, sex, and subsites within colorectum. The beneficial effect of dietary methionine intake on CRC risk appeared limited to the studies with a longer duration of follow-up (the random-effects model RR = 0.81, 95% CI = 0.70 - 0.95), to the Western populations (the random-effects model RR = 0.83, 95% CI = 0.73 - 0.95), to men (the random-effects model RR = 0.75, 95% CI = 0.57 - 0.99), and to colon cancer (the random-effects model RR = 0.77, 95% CI = 0.64 - 0.92). When we limited the analysis to the four studies [12,15-17] that followed up for >10 years, women with highest methionine intake had 14% (the random effects model RR = 0.86, 95% CI = 0.74-1.01) borderline non-significant (*P* for test = 0.06) lower risk of CRC. 

**Table 1:** Meta-analysis of risk estimates of dietary methionine intake (highest versus lowest) and risk of incident colorectal cancer.

	Number of studies	References	RR (95 % CI)		Heterogeneity test	
			Random-effect model	Fix-effects model	*P*	*I* **^*2*^** (%)
All studies	8	12-18	0.89 (0.77-1.03)	0.89 (0.79-1.00)	0.18	29.1
Follow-up						
＞10 years	5	12,15,16,17	0.81 (0.70-0.95)	0.82 (0.71-0.94)	0.33	13.6
＜10 years	3	13,14,18	1.08 (0.87-1.34)	1.08 (0.87-1.34)	0.48	0.0
Number of cases						
＞500	4	12,14-16	0.89 (0.74-1.08)	0.89 (0.76-1.03)	0.24	26.0
＜500	4	13,17,18	0.89 (0.69-1.15)	0.89 (0.75-1.07)	0.12	49.4
Areas						
Asian	2	14,18	1.19 (0.90-1.57)	1.19 (0.90-1.57)	0.53	0.0
Western	6	12,13,15-17	0.83 (0.73-0.95)	0.83 (0.73-0.95)	0.40	3.9
Sex						
Men	3	12,14,17	0.75 (0.57-0.99)	0.75 (0.58-0.97)	0.31	15.2
Women	7	12-18	0.94 (0.80-1.09)	0.93 (0.82-1.06)	0.23	26.3
Subsites						
Colon	4	12,17,25	0.77 (0.64-0.92)	0.77 (0.64-0.92)	0.58	0.0
Rectal	2	12,25	0.88 (0.55-1.42)	0.89 (0.58-1.38)	0.31	15.3

RR, relative risk; CI, confidence interval.

**Figure 3:**
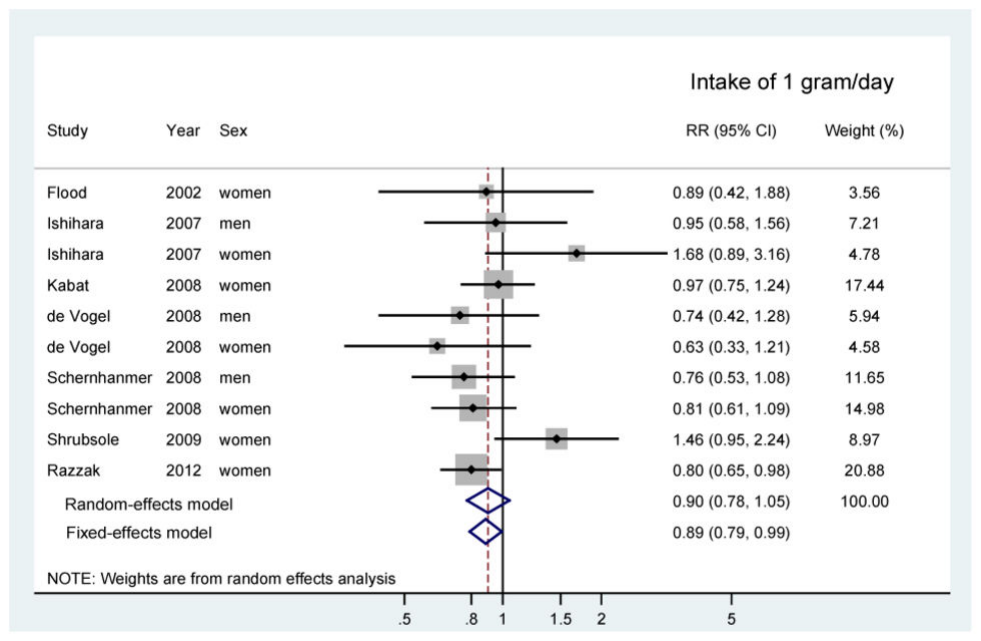
Forest plot showing relative risks of incident colorectal cancer for an increase in dietary methionine intake of 1 mg/day for individual studies and all studies combined. RR, relative risk; CI, confidence interval.

The results of the dose-response meta-analysis also suggested an inverse association between dietary methionine intake risk of CRC, though only the results based on the random-effect model was not statistically significant (Figure 3). 

**Figure 4:**
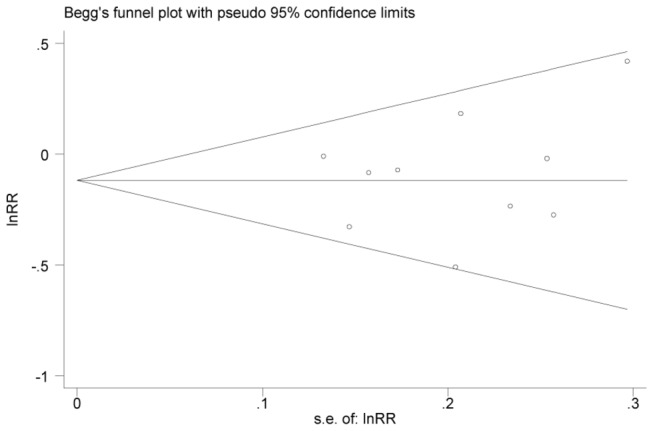
Begg’s funnel plot with pseudo-95% confidence limits for the relative risks of colorectal cancer and dietary methionine intake (highest compared with lowest category of intake).

There was no indication of publication bias from either visualization of the funnel plot (Figure 4) or Egger’s test (*P* for Egger’s test = 0.63).

## Discussion

To the best of our knowledge, this is the first meta-analysis of the prospective association between dietary methionine intake and risk of incident CRC. Results from this large analysis including 6,331 CRC cases and 431,029 participants suggest that dietary methionine intake may be associated with reduced risk of CRC, especially colon cancer. 

In the subgroup analysis, a significant inverse association was observed when pooling studies with a follow-up period of at least 13.3 years, but no association was found when combining those with a shorter duration. Several possibilities may explain this finding. Clearly, studies with shorter follow-up have had lower number of cases and less power to detect associations. Moreover, both two Asian studies, which appeared to be outliers (reported RRs of ＞1), followed up for less than 10 years, and so the difference by follow-up time may have occurred by chance. However, this finding might also suggest that the latency time between methionine intake and the clinical detection of CRC might serve as an effect modification of the association. Recent evidence from large prospective studies documents that total folate intake in the remote past (12-16 years before diagnosis), rather than close to diagnosis (0-8 years) was associated with reduced risk of CRC; furthermore, the investigators also found that intake close to diagnosis (0-8 years) was most strongly associated with lower risk of colorectal adenoma, indicating that folate intake may be beneficial only during early preadenoma stages [33]. Both methionine and folate are the key components of one carbon metabolism. Therefore, it is possible that the protective effect of methionine may also be restricted to the initiation or early development of CRC. In fact, prospective epidemiologic evidence has found a significant inverse relationship between dietary methionine intake and risk of colorectal adenomas [34]. Given the long progression time of adenomas that develop into cancers [35], studies with a short follow-up period may not be expected to identify additional benefits of methionine intake on CRC development.

There are several mechanisms whereby dietary methionine intake may reduce the risk of CRC. Mehionine is an important naturally occurring biomolecule found in all mammalian cells, and is required for the synthesis of *S*-adenosylmethionine (SAM), which is the primary methyl donor for methylation process [36,37]. In experimental study, SAM induces apoptotic cell death in colon cancer cell [38]. Animal studies demonstrate that SAM reduces inflammation-induced colon cancer and inhibits several pathways that are important in colon carcinogenesis [9]. Furthermore, methionine synthase polymorphisms have also been found to be involved in the development of CRC [39]. 

This meta-analysis has several strengths. Limiting the analysis to prospective studies could eliminate the possibility of recall bias and selection bias that are universal in the retrospective case-control studies, and including more than six thousand CRC cases also minimizes the possibility that the observed findings are chance results. 

We have also acknowledged that this study has several limitations. First, we cannot resolve uncontrolled confounders as a potential explanation for the observed association, because a meta-analysis of observational studies cannot exclude residual or unknown confounders that could be inherent in the original studies. For example, individuals with higher dietary methionine intake may be at lower risk of CRC due to other health habits. However, this likelihood was largely reduced by the multivariable adjustment of included studies. Most included studies were controlled for age, BMI, smoking, alcohol drinking, physical activity and intakes of energy, calcium and meat. Second, diet intake was assessed by a self-administered FFQ in most included studies, and all but two [17] of included studies used a single measurement of baseline diet intake. Therefore, potential changes in dietary habits over the long follow-up period cannot be accounted for, resulting in misclassification of exposure. In cohort studies, misclassification is generally nondifferential, which leads to null rather than to spurious associations. Third, there is a wide range of values for the cutoff points for the highest and lowest categories for dietary methionine intake in different studies, which might also affect the current analysis. Finally, since this meta-analysis was based on the published literature, publication bias is always a concern. However, we found no evidence of such bias in this analysis.

In summary, findings of this meta-analysis suggest that dietary methionine intake may be associated with reduced risk of CRC, especially colon cancer. Future prospective studies with long follow-up durations are needed to confirm these findings. 

## Supporting Information

Table S1 **Characteristics of the included prospective studies.**
(DOCX)
