# Supplementary material for: Dietary Methionine Intake and Risk of Incident Colorectal Cancer: A Meta-Analysis of 8 Prospective Studies Involving 431,029 Participants
Source: PLoS One. 2013 Dec 10;8(12):e83588. doi: 10.1371/journal.pone.0083588 (PMC3858442; doi:10.1371/journal.pone.0083588)
Supplement: Table S1 — Characteristics of the included prospective studies. (DOCX) [file pone.0083588.s001.docx]

| Study | Follow-up, years | Country | Sex | Cases/  subjects | Age, years | Mehionine intake,  highest versus lowest | RR (95 % CI),  highest versus lowest | Adjustment | Methionine intake  assessment |
| --- | --- | --- | --- | --- | --- | --- | --- | --- | --- |
| Flood, 2002 | 8.5 | USA | Women | 490/  45264 | 40-93 | ＞0.91 vs ＜0.58  g/1000 kcal | 0.93 (0.66-1.3) | Intake s of energy, dietary folate, alcohol, calcium, and vitamin D. | Self-administered FFQ |
| Ishihara, 2007 | 5.8 | Japan | Men and women | 526 /  81184 | 45-74 | Men:  2.171 vs 1.245 g/d  Women:  2.065 vs 1.188 g/d | Men:  0.98 (0.60-1.62)  Women:  1.52 (0.85-2.72) | Age, study area, BMI, physical activity, smoking, supplement use, and intakes of alcohol, calcium, vitamin D, and meat. | Self-administered FFQ |
| Kabat, 2008 | 16.4 | Canada | Women | 617 /  49654 | 40-59 | ＞2.48 vs＜1.78 g/d | 0.99 (0.76-1.28) | Age, BMI, smoking, education, menopausal status, oral contraceptive use, hormone replacement therapy, and intake of energy and alcohol. | Self-administered FFQ |
| de Vogel, 2008 | 13.3 | Netherlands | Men and women | 2349 /  4168 | 55-69 | Men:  2.093 vs 1.366 g/d  Women:  1.841 vs 1.154 g/d | Men:  0.79 (0.50-1.25)  Women:  0.76 (0.46-1.26) | Age, BMI, smoking, family history of CRC, and intakes of alcohol, energy, meat, fat, fiber, calcium, folate, riboflavin, vitamin B-6 and iron. | Self-administered FFQ |
| Schernhammer, 2008 | 18 | USA | Men | 277 /  47371 | 40-75 | ≥2.55 vs ≤1.81 g/d | 0.60 (0.40-0.89) | Age, BMI, screening sigmoidoscopy, family history of CRC, smoking, physical activity, history of colon polyps, multivitamin use, aspirin use, and intakes of energy, beef, calcium, folate, vitamin B6, B12 and alcohol. | Self-administered FFQ |
| Schernhammer, 2008 | 22 | USA | Women | 389/  88691 | 34-59 | ≥2.21 vs ≤1.50 g/d | 0.92 (0.68-1.26) | Age, BMI, screening sigmoidoscopy, family history of CRC, smoking, physical activity, history of colon polyps, multivitamin use, aspirin use, and intakes of energy, beef, calcium, folate, vitamin B6, B12 and alcohol. | Self-administered FFQ |
| Shrubsole, 2009 | 9 | China | Women | 394/  72861 | 40-70 | 2.07 vs 1.26 g/d | 1.2 (0.8-1.8) | Age, BMI, educational, income, smoking, physical activity, hormone replacement therapy, menopausal status, family history of CRC, NSAIDs use, vitamin B supplement, history of colorectal polyps or diabetes, and intakes of energy, vegetables, fruits, red meats, and calcium and alcohol | Interview-based FFQ |
| Razzak, 2012 | 18 | USA | Women | 1289/  41836 | 55-69 | ≥2.23 vs ≤1.36 g/d | 0.72 (0.54-0.96) | Age, BMI, waist-to-hip ratio, smoking, exogenous estrogen use, physical activity, history of diabetes, and daily intakes of total energy, total fat, sucrose, red meat, calcium, vitamin E and alcohol. | Self-administered FFQ |

**Table S1**. Characteristics of the included prospective studies.

BMI, body mass index; CRC, colorectal cancer; FFQ, Food-frequency questionnaire; NSAIDs, nonsteroidal anti-inflammatory drug; RR, relative risk; CI, confidence interval.
